# After the Tsunami: Legal Implications of Mass Burials of Unidentified Victims in Sri Lanka

**DOI:** 10.1371/journal.pmed.0020185

**Published:** 2005-06-28

**Authors:** Clifford Perera

## Abstract

Perera discusses how the tsunami highlighted gaps in the laws in Sri Lanka on death investigation and mass burials.

**Figure:**
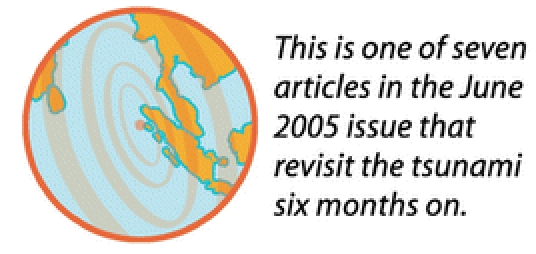


On 26 December 2004, Sri Lanka was affected, along with many other countries in the region, by deadly tsunami waves. The estimated death toll in Sri Lanka was more than 40,000; many more thousands of people were missing and displaced. Sri Lanka had not experienced a disaster of such magnitude in its 2,000 years of recorded history. The administrative, health, and judicial services were simply not able to respond rapidly to the workload demands created by the disaster.

The disaster itself also highlighted gaps in the laws on death investigation and mass burials, which required the rapid introduction of many emergency regulations and modification of existing laws. This article discusses these gaps, and categorises the post-tsunami events into two phases: acute and secondary. The acute phase concerns the events that occurred during the first week with regard to the management of the deceased; the secondary phase represents the events that took place subsequently.

## Legal Provision for a Death Investigation System

Death investigation is an essential process to maintain law and order in any civilized society. Sri Lanka adopted the Coroner system from Britain in the 18th century, along with other South Asian countries, and still uses it, with some modifications. The legal provisions for death investigation are elaborated in Sections 369–373 in the Code of Criminal Procedure, Act 15, of 1979 of Sri Lanka (available at http://www.lawnet.lk). These sections do not, however, provide any instructions with regard to a mass disaster situation. Under the present system, death investigation is principally carried out by an Investigator into Sudden Death appointed by the Minister of Justice for a particular jurisdiction. Unlike in European countries, most of these investigators have neither medical nor legal professional skills. On certain occasions, such as homicides and custodial deaths, the magistrates may act as investigators of death.

When emergency laws are activated, the usual death investigation procedures may deviate from accepted norms, and the authority for disposal of bodies—even without post-mortem examinations—has been given in the past to prescribed police authorities. Many such emergency regulations were enacted during the secondary phase of the tsunami in order to meet socio-political requirements. However, there were no specific regulations that related to death investigation or disposal of the deceased.

## Death Investigation System after the Tsunami

In a disaster situation, it is very difficult to follow usual death investigation procedures, especially if the numbers of deceased are very high. Hence, it was not possible to follow usual procedures of death investigation in any of the countries affected by the tsunami in December 2004.

Investigators into Sudden Death and magistrates in the tsunami-affected areas were joined by their counterparts in non-affected areas in performing inquests into the deaths of the deceased. However, many deficiencies were observed in the formal procedure of death investigation and certification, mainly due to lack of descriptive guidelines to follow in a mass disaster situation.

## Identification of the Deceased: Acute Phase

The most important aspect of mass disaster investigation, from the medico-legal perspective, is the identification of the deceased. The cause of death of the deceased is of secondary importance in these circumstances. The importance of the issue of identification of the deceased after the tsunami was brought to light by human rights groups in the early stages. Radhika Coomaraswamy, chairperson of the Human Rights Commission of Sri Lanka, said: “The identification of dead bodies is one of the most basic of all human rights. Due to the situation, many bodies were buried without identification. It is absolutely essential that forensic expertise is marshalled to identify all dead bodies so that their next of kin may be informed. The government must make this an important matter of priority” [1].

However, medico-legal services were not given priority during the acute phase management of the tsunami disaster in Sri Lanka, despite Sri Lanka having the best medico-legal services of the countries affected. The number of qualified full-time forensic pathologists in Sri Lanka is almost equal to that of Australia, although infrastructure facilities are underdeveloped in most centres.

After the tsunami, the deceased were sent to the nearest hospital morgues during the initial stages, and within hours all available space was occupied. After the second day of the disaster, the deceased were sent to mass burial sites, bypassing the hospitals. As a result, thousands of deceased were neither imaged nor documented in appropriate registries before being sent to mass burial grounds. The reasons for this administrative decision are shown in Box 1.

**Box 1. Reasons Why the Deceased Were Neither Imaged nor Documented before Being Sent to Mass Burial Grounds**

- Minimal recognition given to the medico-legal services at the initial stages
- Rapid recovery of dead during first few days
- Lack of storage facilities for the deceased in hospitals
- The advanced state of decomposition observed in the deceased
- Lack of rapid documentation facilities (e.g., digital cameras, computers)
- Minimal use and construction of temporary mortuary spaces
- Lack of transport facilities to bring deceased into hospitals
- Poor coordination amongst forensic pathologists
- Lack of support staff to continue medico-legal work

As most of the statistics on the mass burials were not reliable nor available on demand, the total number of people who died in each region in the acute phase was calculated from hospital data, which were not representative of the true figure. If during the acute phase the deceased had at least been imaged and documented by an organised team of forensic experts in the few hours before mass burials, the overall picture would have been very different.

## Legal Consequences of Mass Burials

The rapid disposal of the deceased into mass burial sites without any sort of documentation had serious effects on issuing death certificates subsequently. Many mass burial sites were not planned and not well documented. The police figures about the deceased in many burial sites were contradictory. In some instances, the identified and unidentified deceased were buried together. In none of the sites were the buried bodies given any permanent identification marks or tags, which would have been helpful for future possible exhumations. In addition, many burial sites were used for reburials.

The system established to collect information on missing persons functioned poorly. The police and courts maintained separate offices for this purpose, and much of their information was duplicated. Therefore, the credibility of the official statistics on missing persons is questionable. Some victims were recorded missing and deceased at two different centres; in other cases, coroners were reluctant to certify death due to lack of vital information.

The provisions of the Legal Aid Commission Act of 1978 should be adopted to ease the problem of lack of death certificates. A special mechanism will need to be derived that can authorise the issuing of death certificates on missing people after an agreed-upon brief period of search by the authorities. During the aftermath of the tsunami, the police, coroners, and courts were acting without coordination. Because of irregularities affecting identification of the deceased and the issuing of death certificates, there remains the possibility of the public raising the issue of their fundamental rights being denied. Such public protest may be driven by the many exhumations performed in January–March 2005 to identify missing foreigners in various parts of southern Sri Lanka.

## Identification of the Deceased: Secondary Phase

**Figure 1:**
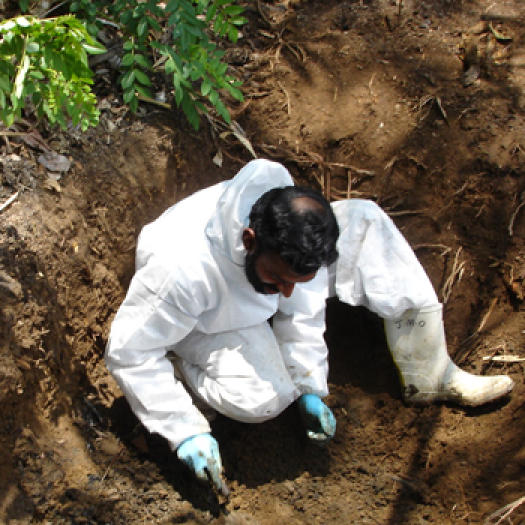
Exhumations Performed in the Southern Province in Search of Missing Foreigners (Photo: Clifford Perera)

**Figure 2:**
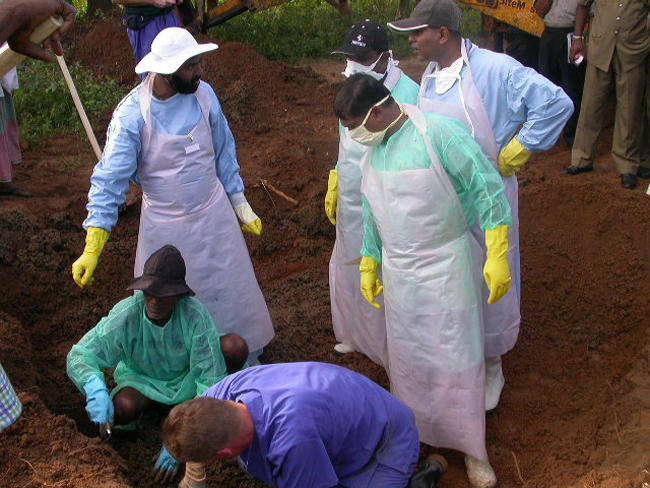
More Exhumations Performed in the Southern Province in Search of Missing Foreigners (Photo: Clifford Perera)

The secondary phase of the disaster was dominated by two events: recovery of human remains from lowlands and seashore, and exhumation of suspected mass burial sites to search for missing foreigners (Figures 1 and 2).

Although the acute phase was carried out with much enthusiasm and with the participation of many segments of civil society, the secondary phase lacked momentum due to the lack of interest among law enforcement authorities and some forensic practitioners. The strain of the increased workload during the first few weeks, lack of coordination amongst the senior law enforcement and medico-legal officials, and lack of infrastructure facilities led to slow continuation of the secondary phase since January 2005. Some of the human remains recovered in this period, even within eight weeks of the disaster, are almost skeletalised—for example, those from marshy lands in the southern province collected within eight weeks. The high temperatures of 30–35 °C prevailing for weeks after the disaster may have had a direct effect on the speed of decomposition. Most of the deceased recovered during the second phase were females and children without any specific identification details.

An international commission was formed in mid-February to identify missing foreigners in Sri Lanka, with the participation of British, German, and French investigators, the officers of the Criminal Investigation Department, judicial medical officers from Colombo and Galle, and a coroner in Colombo. This commission undertook the task of continuing all the previous investigations started in the search for missing foreigners, including exhumations already performed in many parts of the country. The distinguishing feature of the commission's involvement was performing complete autopsy examinations and identification procedures on all suspected bodies of missing foreigners. This commission functioned until April, and many foreigners were positively identified following secondary (specific) investigations such as DNA profiling. The formation of such a commission led local experts to re-evaluate their strategies in disaster management, and many voices were raised demanding urgent attention to establish proper investigative mechanisms in the state sector, including DNA-profiling facilities to identify the deceased in disasters.

Local experts are realising that the secondary phase of identification may continue for months, despite having minimal resources and innovation for such an exercise. The seabed surrounding the affected coastal regions contains innumerable deceased, most of whom may never be seen or touched. However, the human remains recovered from the beaches of these affected regions must be treated as potential tsunami deceased for many months to come.

## Implications for the Future

Sri Lanka has experienced many man-made disasters, such as bomb explosions, and natural disasters, such as regional floods, in the past three decades. Currently, many countries in Asia have their own disaster management and emergency plans; a few countries such as Sri Lanka lag far behind in modern disaster management. Therefore, it is crucial that Sri Lanka formulate a national plan for disaster management, including national guidelines for disaster victim identification, to overcome the problems discussed in this article.

Previously, Sri Lanka had enacted an act on a reconstruction and rehabilitation fund (Reconstruction and Rehabilitation Fund Act, No. 58, in 1993). A bill called “Sri Lanka Disaster Counter-Measures” was published in the government gazette issued in November 2002 and placed on the order paper of the parliament. The Supreme Court made a determination of the bill in January 2003 after its legality was challenged, but it was not taken for debate in the parliament afterwards. However, this time the Sri Lankan government introduced more or less the same bill under the title “Sri Lanka Disaster Management” in February 2005, two months after the disaster. The long title of the bill states that it is “to provide for the establishment of the National Council for Disaster Management, the National and Human Disaster Management Centre; the appointment of Technical Advisory Committees; the preparation of Disaster Management Plans; the declaration of a state of disaster, the award of compensation, and for matters connected thereto.”

Further, this bill contains the first legal definition of a disaster in Sri Lanka and also includes a separate definition for human disaster (man-made disaster). Section 10 of the bill states that every ministry, government department, and public cooperation prepare a disaster management plan—a long-awaited request. The framework suggested by this bill for disaster management in Sri Lanka is commendable and is an effort to uplift the local standards to the existing regional and international standards through a strong legal framework. Although the bill does not specify a time frame to establish disaster management structures, the legal implications of it will drastically affect the management of future disasters.

In its determination in 2005, the Supreme Court was critical of the failures of the previous parliament and reiterated the necessity of implementing relevant legal infrastructure: “We have to note that if the Bill in respect of which the determination was made by this court in January 2003 was proceeded with in parliament, a National Disaster Management plan would have been in place to cover natural disasters including a ‘tsunami.’ For some reason the Bill was not proceeded with by Parliament, and the court has once again to make a determination on substantially the same bill, two years after a devastating ‘tsunami’ has affected Sri Lanka, at a time when the country was totally unprepared to meet that situation” [2].

## Conclusion

It is clearly a priority for Sri Lanka to have an effective medico-legal scheme to deal with problems of identification of deceased in future disasters. It is a collective responsibility of the attorney general's department, health department, police department, administrative authorities, and professional organisations of forensic pathologists, scientists, legal practitioners, and many others concerned to ensure that this happens.
